# Is a history of work-related low back injury associated with prevalent low back pain and depression in the general population?

**DOI:** 10.1186/1471-2474-9-22

**Published:** 2008-02-19

**Authors:** Cesar A Hincapié, J David Cassidy, Pierre Côté

**Affiliations:** 1 Centre of Research Expertise in Improved Disability Outcomes (CREIDO), University Health Network, Toronto, Canada; 2 Division of Health Care and Outcomes Research, Toronto Western Research Institute, Toronto, Canada; 3 Department of Public Health Sciences, Faculty of Medicine, University of Toronto, Toronto, Canada

## Abstract

**Background:**

Little is known about the role of prior occupational low back injury in future episodes of low back pain and disability in the general population. We conducted a study to determine if a lifetime history of work-related low back injury is associated with prevalent severity-graded low back pain, depressive symptoms, or both, in the general population.

**Methods:**

We used data from the Saskatchewan Health and Back Pain Survey – a population-based cross-sectional survey mailed to a random, stratified sample of 2,184 Saskatchewan adults 20 to 69 years of age in 1995. Information on the main independent variable was gathered by asking respondents whether they had ever injured their low back at work. Our outcomes, the 6-month period prevalence of severity-graded low back pain and depressive symptoms during the past week, were measured with valid and reliable questionnaires. The associations between prior work-related low back injury and our outcomes were estimated through multinomial and binary multivariable logistic regression with adjustment for age, gender, and other important covariates.

**Results:**

Fifty-five percent of the eligible population participated. Of the 1,086 participants who responded to the question about the main independent variable, 38.0% reported a history of work-related low back injury. A history of work-related low back injury was positively associated with low intensity/low disability low back pain (OR, 3.66; 95%CI, 2.48–5.42), with high intensity/low disability low back pain (OR, 4.03; 95%CI, 2.41–6.76), and with high disability low back pain (OR, 6.76; 95%CI, 3.80–12.01). No association was found between a history of work-related low back injury and depression (OR, 0.85; 95%CI, 0.55–1.30).

**Conclusion:**

Our analysis shows an association between past occupational low back injury and increasing severity of prevalent low back pain, but not depression. These results suggest that past work-related low back injury may be an important risk factor for future episodes of low back pain and disability in the general population.

## Background

Low back pain (LBP) is a significant public health problem in all industrialized nations. It is associated with considerable disability, healthcare use, and societal costs. It affects 60% to 85% of all people in their lifetime, and between 15% to 30% on any given day [1,2]. Although most episodes of LBP are mild, more than 10% of the population experience disabling LBP during any 6-month period [3].

Contrary to prior belief, most people with LBP do not experience complete, permanent recovery of their condition. In Saskatchewan, less than 30% of individuals with prevalent LBP experience resolution of their pain over a 12-month period, and of those who do experience resolution, more than 20% will report a recurrent episode within 6 months [4]. Moreover, Pengel and colleagues have systematically reviewed this literature and found that rapid improvements occur in the first 3 months after onset, but that improvements are gradual thereafter [5]. In another review, Hestbaek and colleagues conclude that LBP is not a self-limiting condition [6]. After 6 months of follow-up, between 3% and 40% of patients initially off-work remain off-work, while at 12 months post-onset, over 40% of people with LBP report persistent pain. Recurrences of work absence due to LBP are quite common, with about one third of people reporting recurrent work absence within 12 months of LBP onset [6]. The high economic and social burden associated with LBP is thus mainly attributable to a small proportion of individuals with recurrent and persistent disabling pain.

The issue of disabling LBP attributable to work-related low back injury is very relevant to workers, employers, workers' compensation insurers, healthcare providers, and society in general. However, the etiology of LBP is still not well understood. Much of the epidemiologic literature on risk factors for LBP has focused on specific occupational groups [7-11]. However, these types of studies on selected populations of workers are vulnerable to the "healthy worker effect" [12]; a self-selection bias that allows relatively healthy people to remain in certain jobs, whereas those who change jobs are as a group less healthy [13]. Nonetheless, it is generally accepted that the etiology of LBP is multifactorial and related to a variety of factors such as individual, occupational, psychologic, as well as lifestyle characteristics [14-19]. Although it is well documented that a history of LBP is a predictor of future pain and disability in workers [20,21], much less is known about the severity of pain and disability experienced by these individuals and whether a history of work-related low back injury is an important factor in disabling LBP in the general population. Furthermore, prior low back injury is a distinct yet related construct to prior LBP and may have a different association with future disabling LBP and depression.

A 1995, representative, population-based survey from Saskatchewan, Canada, provided an opportunity to examine if a lifetime history of work-related low back injury is associated with prevalent severity-graded LBP, depressive symptomatology, or both, in the general population.

## Methods

### Study design and population

Our data are from the Saskatchewan Health and Back Pain Survey (SHBPS) – a population-based cross-sectional mailed survey of the distribution and factors associated with spinal disorders and depression in the province of Saskatchewan. The survey was conducted in September 1995. Saskatchewan is a Canadian province with a universal health care system that at the time of data collection had a population of 1,021,180 inhabitants.

The target population for the SHBPS consisted of 593,464 residents between the ages of 20 to 69 years who held a valid Saskatchewan Health Services card on August 31, 1995. Excluded from the target population were inmates of provincial correctional facilities, residents under the Office of the Public Trustee (mentally handicapped and severely disabled), foreign students and workers holding employment or immigration visas, and residents of special care homes.

From the target population a weighted, age-stratified random sample of 2,184 residents was formed using the Saskatchewan Health Insurance Registration File (HIRF) as the sampling frame. The HIRF provided more than 99% coverage of the Saskatchewan population at the time and contained basic demographic data that allowed the representativeness of the study sample and the impact of nonresponse bias to be assessed. The selection strategy and sample size estimate for the survey have been previously described in detail [3,22]. Participation in the survey was voluntary. The University of Saskatchewan Advisory Committee on Ethics in Human Experimentation approved the survey, and this analysis was reviewed by the University Health Network Research Ethics Board.

### Survey participation

The age-stratified random sample of the HIRF provided a sample representative of the Saskatchewan population in terms of age, gender, and location of residence [3]. After three mailing waves, 1,133 questionnaires were returned for a cumulative response proportion of 55.1%. Two questionnaires were completed by subjects outside of the predetermined age range resulting in a final study sample of 1,131 participants, or 55% of the sample population. Survey participation was associated with being older, female, married, and not residing on a First Nations Reserve [3].

### Main independent variable: history of work-related low back injury

The SHBPS measured whether respondents had a lifetime history of work-related low back injury by asking, "Have you ever injured your low back at work?" We classified as exposed those persons who answered, "Yes" to this question. As a check on misclassification in our measure, we assessed responses to the following supplementary question, "Have you ever had to take time off work or perform light duties at work because of a low back work injury?" Of 1,093 respondents who responded to this question, 272 (24.1%) answered "Yes." All respondents who indicated having taken time off work or performed light duties at work due to a work injury reported a history of work-related low back injury in responding to our main independent variable.

### Outcomes

#### Severity-graded LBP

A body diagram was used to explicitly identify the location of LBP in the SHBPS [3]. LBP was defined as pain located between the 12^th ^ribs and the gluteal folds experienced in the previous 6 months. The main outcome in this study is the 6-month period prevalence of graded severity of LBP, as measured by the Chronic Pain Questionnaire (CPQ) [23]. The questionnaire is a seven-item Guttman scale that was developed to classify pain into grades of severity [24]. Three items assess pain intensity and four items assess disability over the previous 6 months. Pain intensity is rated according to the following variables: i) today's pain, ii) the worst pain in the last 6 months, and iii) the average pain experienced in the last 6 months. Three disability questions measure the interference over the past 6 months caused by pain with respect to: work; recreational, social, and family activities; and, daily activities. The fourth disability question measures the number of days in the past 6 months that the respondent has been kept from usual activities (work, school, or housework). Five grades of pain severity are derived from the three disability scores and the number of disability days, and are calculated based on the aggregate score of pain intensity (ranging from 0–100) and the number of disability points (ranging from 0–6) (Table 1). In this study, because the number of participants with Grades III and IV LBP was small, we combined these into one category, Grade III-IV LBP (that is, high-disability LBP).

**Table 1:** Classification of low back pain grade

Grade	Scoring	Interpretation
0	No pain, no disability	No chronic pain
I	PI < 50; DP <3	Low pain intensity/low disability
II	PI ≥ 50; DP < 3	High pain intensity/low disability
III	DP = 3–4	High disability/moderately limiting
IV	DP = 5–6	High disability/severely limiting

Abbreviations: PI, pain intensity; DP, disability points

The CPQ has been shown to be a reliable and valid measure in various pain populations [25-29]. The pain grades show a statistically significant and monotonically increasing relationship with employment status (full employment to unemployment), amount of pain-related functional limitation, increasing levels of depression, and decreasing levels of self-rated health [23,24].

#### Depression

The Center for Epidemiological Studies-Depression Scale (CES-D) was used to measure the 1-week period prevalence of depression. The CES-D is a widely used 20-item self-report scale designed to measure current level of depressive symptoms in population-based epidemiologic research [30]. It has been shown to be a valid and reliable measure in various populations, including the general community, primary care settings, and arthritis and chronic pain populations [31-34]. The CES-D is scored from 0 to 60. A score of 16 or higher is considered indicative of major depression in the general population [30,35].

### Covariates

The SHBPS questionnaire included several valid and reliable inventories and specific questions inquiring about various domains:

#### Sociodemographic characteristics

Information was collected on age, gender, marital status, location of residence, as well as highest education attainment and annual household income.

#### Work-related characteristics

Data on employment status and main work activity was gathered. For work activity, participants' were asked to check one main activity from the following options: "heavy labour," "light labour," "mostly sitting," "mostly standing," or "mostly walking/moving around."

#### General health

The Medical Outcomes Study 36-item short form questionnaire (SF-36) was used to measure current self-perceived general health status [36]. The SF-36 is designed to provide a global measure of health-related quality of life. The SF-36 is a valid and reliable measure that has been tested extensively in various populations [37-39]. For the purpose of this analysis, the general-health scale was used as a continuous variable with scores ranging from 0 to 100, with higher scores suggesting better health status.

#### Comorbidities

Coexisting health problems may be covariates with important effects on the associations between a history of work-related low back injury and both, prevalent LBP and depressive symptoms. A self-report questionnaire was developed inquiring about the presence and perceived impact of broad categories of health disorders and included in the SHBPS. Participants were asked if they currently experience a condition. If so, participants were then asked to evaluate the impact of the condition on their health over the past 6 months. The severity was measured on a modified Likert scale, with answers graded from: "does not affect my health," to "makes my health a little worse," "makes my health worse," or "makes my health much worse" than it should be. This severity grading was used for the purposes of the analysis. The Comorbidity Questionnaire has been shown to have good test-retest reliability and adequate face and concurrent validity [40,41]. The comorbidities of interest were allergies, arthritis, breathing problems, cardiovascular disorders, digestive problems, high blood pressure, mental health problems, and headaches. In addition, the Chronic Pain Questionnaire was used to measure the presence and severity of neck pain experienced in the previous 6 months.

#### Cigarette smoking

Self-report of smoking status was obtained and categorized as nonsmoker, ex-smoker or current smoker.

#### Anthropometric variables

Height and weight were used to compute the body mass index (BMI; BMI = kg/m^2^).

#### Exercise

Data on the average number of days per week participating in a minimum of 30 minutes of exercise during the previous 6 months was collected.

#### Psychosocial

Participants were asked about their current level of satisfaction with their life. Possible responses included: "very satisfied," "satisfied," "neither satisfied nor dissatisfied," "dissatisfied," and "very dissatisfied."

### Statistical analysis

Descriptive statistics were used to describe the distribution and associations of the variables in those respondents who reported a history of work-related low back injury and in those who did not. We built logistic regression models to test and measure associations between a lifetime history of work-related low back injury and the two outcomes while controlling for potential covariates [42]. Multinomial logistic regression analysis was used for the models investigating severity-graded LBP, while binary logistic regression analysis was used for depressive symptomatology. We measured the strength of associations with odds ratios (ORs), and the precision of these estimates were given by their 95% confidence intervals (CIs). The analysis was conducted using SAS [43,44].

A three-step modeling approach was used to determine the effects of covariates on the associations of interest. First, we calculated the crude ORs and 95% CIs for a lifetime history of work-related low back injury. Second, we individually built bivariate models that included the main independent variable and each of the potential covariates to assess their effect on the crude association. When they were not the outcome being analyzed, severity-graded LBP and depressive symptomatology were also considered potential covariates in the model. A cut-point of 10% change in the association estimate was considered important enough to include the variable as a covariate in the adjusted model [13]. Finally, to assess the aggregate effect of covariates on the association estimate, all covariates identified in the second step were entered in the final model. In the final model, the covariates that did not contribute to the model (*p *≥ 0.1 on the Wald χ^2^-statistic) were excluded and their impact on the association estimate evaluated. If the estimate did not vary by more than 10% in the absence of that variable, it was not considered an important covariate in the final model. The final models were also adjusted for age and gender. At each stage of modeling, numerical problems (zero cell count or covariates discriminating the outcome perfectly) were assessed by comparing the estimated standard error to the point estimate. The presence of a large standard error relative to the point estimate was considered suggestive of a numerical problem [45]. The variables responsible for numerical problems were excluded from the model.

#### Supplemental analysis

Due to concern that our main independent variable might result in non-differential misclassification of the responders, we complemented our main analyses with similar analyses using a supplementary independent variable measuring lifetime history of time off work or light duties due to work-related low back injury.

## Results

**Figure 1:**
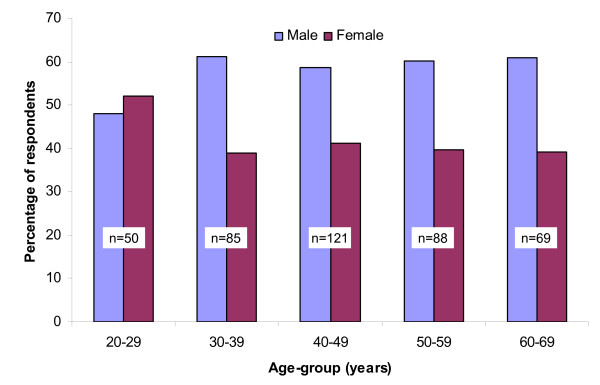
Age-group and gender specific distribution of lifetime history of work-related low back injury (*n *= 413).

Of the 1,131 eligible participants, 1,086 responded to the question about the main independent variable. Of these, 38.0% (413/1,086) reported a positive history of work-related low back injury. A history of work-related low back injury was more common in men than in women from all age groups except the 20- to 29-year-old age group, where the proportions of men and women with a history of injury were similar (Figure 1). The characteristics of the study sample stratified by history of low back injury at work and the supplementary variable, history of time off or light duties due to a work-related low back injury, are presented in Tables 2, 3, 4. A higher proportion of respondents with a history of work-related low back injury lived in rural Saskatchewan and a higher percentage also reported an annual household income of less than $40,000 (Table 2). In addition, a lower proportion of previously injured respondents had at least some post-secondary education.

**Table 2:** Frequency distribution of the sociodemographic and work-related characteristics by main and supplementary independent variables.

Characteristic	History of work-related low back injury		History of time off or light duties due to work-related low back injury	
	
	Yes	No	Yes	No
Age – mean (SD)	45.6 (12.6)	43.9 (13.5)	46.7 (12.5)	43.8 (13.4)
Male gender – N (%)	242 (58.6)	267 (39.7)	169 (62.1)	339 (41.9)
Marital status – N (%)				
Married/Common law	312 (76.3)	498 (74.7)	205 (76.2)	604 (75.2)
Divorced/Separated	37 (9.1)	46 (6.9)	24 (8.9)	57 (7.1)
Widowed	8 (2.0)	21 (3.2)	7 (2.6)	22 (2.7)
Single	52 (12.7)	102 (15.3)	33 (12.3)	120 (14.9)
Location of residence – N (%)				
Urban	135 (32.8)	259 (38.5)	85 (31.4)	308 (38.1)
Rural	277 (67.2)	413 (61.5)	186 (68.6)	501 (61.9)
Annual household income – N (%)				
0 – 20,000	103 (26.3)	126 (20.5)	68 (26.5)	160 (21.5)
20,001 – 40,000	126 (32.2)	219 (35.6)	85 (33.1)	258 (34.6)
40,001 – 60,000	87 (22.3)	142 (23.1)	59 (23.0)	169 (22.7)
60,001 and over	75 (19.2)	128 (20.8)	45 (17.5)	158 (21.2)
Education – N (%)				
Grade 8 or less	38 (9.3)	37 (5.6)	26 (9.6)	48 (6.0)
Some high school	110 (26.8)	133 (20.0)	78 (28.9)	165 (20.6)
High school graduate	107 (26.1)	177 (26.6)	73 (27.0)	209 (26.1)
Some post-secondary	117 (28.5)	213 (32.0)	73 (27.0)	256 (32.0)
University graduate	38 (9.3)	105 (15.8)	20 (7.4)	123 (15.4)
Current employment status – N (%)				
Full-time	212 (52.4)	322 (48.6)	138 (51.7)	396 (49.7)
Part-time	67 (16.5)	97 (14.7)	40 (15.0)	121 (15.2)
Unemployed	31 (7.7)	34 (5.1)	18 (6.7)	47 (5.9)
Homemaker	56 (13.8)	134 (20.2)	36 (13.5)	87 (10.9)
Retired	48 (11.9)	75 (11.3)	37 (13.9)	152 (19.1)
Student	13 (3.2)	31 (4.7)	6 (2.3)	38 (4.8)
Main work activity – N (%)				
Heavy labour	77 (20.0)	49 (7.9)	46 (18.5)	79 (10.5)
Light labour	79 (20.4)	95 (15.4)	52 (20.9)	122 (16.2)
Sitting	62 (16.0)	201 (32.5)	37 (14.9)	226 (29.9)
Standing	42 (10.8)	63 (10.2)	22 (8.8)	82 (10.9)
Walking	138 (35.6)	198 (32.0)	91 (36.6)	245 (32.5)

**Table 3:** Comorbidities in the study population by main and supplementary independent variables.

	History of work-related low back injury		History of time off or light duties due to work-related low back injury	
	
	Yes – N (%)	No – N (%)	Yes – N (%)	No – N (%)
Allergies				
Absent	226 (56.4)	387 (58.6)	144 (54.8)	467 (58.8)
No to minimum impact on health	118 (29.4)	199 (30.2)	76 (28.9)	241 (30.4)
Moderate to severe impact on health	57 (14.2)	74 (11.2)	43 (16.4)	86 (10.8)
Breathing problems				
Absent	266 (66.2)	478 (72.4)	165 (62.7)	576 (72.5)
No to minimum impact on health	95 (23.6)	140 (21.2)	66 (25.1)	168 (21.1)
Moderate to severe impact on health	41 (10.2)	42 (6.4)	32 (12.2)	51 (6.4)
High blood pressure				
Absent	337 (84.0)	560 (84.6)	221 (84.0)	673 (84.6)
No to minimum impact on health	43 (10.7)	74 (11.2)	25 (9.5)	92 (11.6)
Moderate to severe impact on health	21 (5.2)	28 (4.2)	17 (6.5)	31 (3.9)
Cardiovascular disorders				
Absent	339 (83.9)	570 (86.1)	224 (84.5)	682 (85.6)
No to minimum impact on health	48 (11.9)	68 (10.3)	30 (11.3)	86 (10.8)
Moderate to severe impact on health	17 (4.2)	24 (3.6)	11 (4.2)	29 (3.6)
Digestive system problems				
Absent	284 (70.1)	499 (75.3)	176 (66.2)	605 (75.8)
No to minimum impact on health	75 (18.5)	112 (16.9)	53 (19.9)	133 (16.7)
Moderate to severe impact on health	46 (11.4)	52 (7.8)	37 (13.9)	60 (7.5)
Headaches				
Absent	158 (39.0)	314 (47.4)	98 (36.8)	373 (46.7)
No to minimum impact on health	157 (38.8)	248 (37.4)	103 (38.7)	301 (37.7)
Moderate to severe impact on health	90 (22.2)	101 (15.2)	65 (24.4)	124 (15.5)
Arthritis				
Absent	257 (64.4)	507 (77.6)	159 (60.9)	603 (76.6)
No to minimum impact on health	73 (18.3)	95 (14.6)	51 (19.5)	116 (14.7)
Moderate to severe impact on health	69 (17.3)	51 (7.8)	51 (19.5)	68 (8.6)
Mental health problems				
Absent	285 (70.4)	526 (79.3)	183 (69.1)	627 (78.5)
No to minimum impact on health	82 (20.3)	101 (15.2)	55 (20.8)	125 (15.6)
Moderate to severe impact on health	38 (9.4)	36 (5.4)	27 (10.2)	47 (5.9)
Neck pain				
Absent	153 (38.2)	341 (51.6)	104 (39.9)	389 (48.8)
Low intensity/low disability	177 (44.1)	235 (35.6)	109 (41.8)	302 (37.9)
High intensity/low disability	47 (11.7)	58 (8.8)	29 (11.1)	75 (9.4)
High disability	24 (6.0)	27 (4.1)	19 (7.3)	31 (3.9)

**Table 4:** Frequency distribution of health-related characteristics and outcomes by main and supplementary independent variables.

Characteristic	History of work-related low back injury		History of time off or light duties due to work-related low back injury	
	
	Yes	No	Yes	No
Smoking – N (%)				
Never smoked	184 (46.1)	352 (54.1)	111 (42.5)	424 (54.0)
Past smoker	94 (23.6)	158 (24.3)	70 (26.8)	180 (22.9)
Current smoker	121 (30.3)	141 (21.7)	80 (30.7)	181 (23.1)
BMI weight categories (kg/m2) – N (%)				
<18.5 (underweight)	17 (4.1)	31 (4.6)	11 (4.0)	37 (4.6)
18.5–24.9 (normal)	150 (36.3)	271 (40.3)	93 (34.2)	325 (40.1)
25.0–29.9 (overweight)	175 (42.4)	258 (38.3)	121 (44.5)	311 (38.4)
≥ 30.0 (obese)	71 (17.2)	113 (16.8)	47 (17.3)	137 (16.9)
General health from SF-36 – mean (SD)	62.8 (13.7)	64.8 (14.0)	61.8 (13.4)	64.8 (14.0)
Days of exercise per week – mean (SD)	2.9 (2.2)	2.7 (2.1)	2.9 (2.2)	2.8 (2.1)
Life satisfaction				
Very satisfied	81 (19.9)	182 (27.5)	47 (17.5)	216 (27.2)
Satisfied	211 (51.8)	327 (49.5)	134 (49.8)	402 (50.6)
Neither satisfied nor dissatisfied	76 (18.7)	102 (15.4)	62 (23.1)	114 (14.3)
Dissatisfied	32 (7.9)	38 (5.8)	25 (9.3)	45 (5.7)
Very dissatisfied	7 (1.7)	12 (1.8)	1 (0.4)	18 (2.3)

Outcome				
Low back pain				
Absent	48 (11.8)	249 (37.7)	30 (11.2)	267 (33.5)
Low intensity/low disability	221 (54.3)	292 (44.2)	133 (49.8)	378 (47.5)
High intensity/low disability	65 (16.0)	68 (10.3)	42 (15.7)	90 (11.3)
High disability	73 (17.9)	51 (7.7)	62 (23.2)	61 (7.7)
Depressive symptomatology				
CES-D score <16	300 (75.8)	511 (80.0)	193 (74.2)	617 (79.9)
CES-D score ≥ 16	96 (24.2)	128 (20.0)	67 (25.8)	155 (20.1)

Similar proportions of respondents with and without a history of low back injury at work reported comorbidities (Table 3). However, those with a history of work-related low back injury were more likely to report neck pain, moderate to severe headaches, and were more than twice as likely to report arthritis that moderately to severely affected their health. Other health-related characteristics were also, for the most part, quite similar between those with a history of low back injury at work and those without (Table 4). Nonetheless, a lower proportion of respondents with a history of work-related injury reported being "very satisfied" with their lives (19.9% vs. 27.5%), and a higher percentage were current smokers at the time of the survey (Table 4).

All outcomes were more common in respondents who reported a history of work-related low back injury than in those who did not (Table 4). Respondents who had experienced a low back injury at work were more than twice as likely to report suffering from disabling low back pain in the 6 months prior to the survey (17.9% vs. 7.7%). The prevalence of depressive symptomatology was also slightly higher in those with a history of work-related low back injury (24.2%) than in those without (20.0%).

Table 5 presents the crude and adjusted ORs and 95% CIs for the associations between a history of work-related low back injury and both, severity-graded LBP and depressive symptomatology. The analysis demonstrates strong, positive associations between a history of low back injury at work and grades of LBP in the previous 6 months. The difference between the crude and adjusted odds ratios was small (Table 5). The depressive symptomatology model suggests that a history of work-related low back injury is not associated with prevalent depressive symptoms over the past week (Table 5). Income, mental health, life satisfaction, and LBP were important covariates in the adjusted model describing the association between a history of work-related low back injury and prevalent depressive symptomatology.

**Table 5:** Crude and adjusted ORs and 95% CIs for the associations between the main and supplementary independent variables and both, severity-graded low back pain and depressive symptoms.

Outcome	Work-related low back injury		Time off or light duties due to work-related low back injury	
	
	Crude OR	Adjusted OR	Crude OR	Adjusted OR
Severity-graded LBP		*		†
No low back pain	1	1	1	1
Low intensity/low disability	3.93 (2.75–5.60)	3.66 (2.48–5.42)	3.13 (2.05–4.80)	2.99 (1.88–4.77)
High intensity/low disability	4.96 (3.13–7.85)	4.03 (2.41–6.76)	4.15 (2.46–7.03)	3.33 (1.85–5.98)
High disability	7.42 (4.63–11.91)	6.76 (3.80–12.01)	9.05 (5.39–15.2)	9.03 (4.86–16.77)
Depressive symptoms		‡		§
CES-D score <16	1	1	1	1
CES-D score ≥ 16	1.28 (0.95–1.73)	0.85 (0.55–1.30)	1.38 (1.00–1.92)	0.75 (0.45–1.25)

* OR adjusted for age, gender, income, arthritis, and neck pain

† OR adjusted for age, gender, income, arthritis, digestive problems, and headaches

‡ OR adjusted for age, gender, income, mental health problems, life satisfaction, and LBP

§ OR adjusted for age, gender, income, general health, breathing problems, digestive problems, headaches, mental health problems, life satisfaction, and severity-graded LBP

### Supplemental analysis

Similar analyses with our supplementary independent variable of history of time off work or light duties due to work-related low back injury revealed very similar findings to those obtained by analysis with our main independent variable (Tables 2, 3, 4, 5).

## Discussion

The purpose of this study was to determine if a lifetime history of low back injury at work is associated with prevalent severity-graded LBP and depressive symptomatology. The results suggest that strong associations exist between a history of work-related low back injury and the 6-month prevalence of severity-graded LBP. Our findings are in line with those of Battié and colleagues [46] who reported that a history of low back disorder was related to future reports of LBP, and with those of Hurwitz and Morgenstern [14] who reported that a history of back trauma was associated with prevalent chronic back disability. To our knowledge, however, this study is the first to investigate the association between a history of work-related low back injury and both, severity-graded LBP and depressive symptomatology in a North American, general population study.

In their review, Dempsey and colleagues [47] suggest that prior injury history is an important personal variable that influences low back pain prognosis and merits further study in the literature. It is well documented that a history of occupational back pain is a risk factor for prevalent and future episodes of low back pain and disability in occupational settings [20,21]. More recent studies have begun to investigate the associations between low back pain and specific physical and psychosocial workplace characteristics [15,48,49]. However, little is known about the role previous work-related injury plays in future episodes of back pain in the general population or how it relates to grades of LBP severity. Our study supports the hypothesis that work-related low back injury is an important factor in increasing severity of prevalent low back pain in the general population.

### Strengths and limitations

Several factors contribute to the validity of our findings. Data were from a large and representative cross-section of the general, Saskatchewan adult population. We were able to obtain information on, and perform extensive multivariable adjustment for, many potential covariates of the associations between our main independent variable and outcomes of interest. We were also able to discriminate between various grades of LBP severity and depressive symptomatology through the use of valid, reliable, and meaningful outcomes. Furthermore, our supplemental analyses gave very similar results and support the findings of the main analyses.

Our study has limitations. An important threat to internal validity in a population-based survey is selection bias. Selection bias refers to the distortion of association or evidence due to systematic differences in characteristics between those who participate in the survey and those who do not. If either the main independent variable or the outcome of interest is associated with any of these characteristics, it is likely that the study population is not truly representative of the target population. Nonrespondents to the SHBPS were more likely to be male, younger, single, and reside on a First Nations Reserve [3]. Since Grade I LBP is more common in younger individuals [3] and it is possible that more men than women experience work-related low back injury, we might have underestimated the strength of associations between occupational low back injury and prevalent severity-graded LBP. Although the data were collected 13 years ago, we think our results remain valid since there does not seem to be any time-related trend in the prevalence of LBP [1,50], nor in the incidence of work-related back injuries claimed in the province of Saskatchewan [51].

Our results cannot be used to infer that a work-related low back injury increases the risk of experiencing future disabling LBP. In cross-sectional research, a prevalence odds ratio should not be seen as a valid measure of relative risk and should only be used to measure the strength of association. There are four main reasons for this. First, the cross-sectional research design does not address the issue of temporality. In other words, we cannot be certain that the work-related injury has in fact occurred prior to the onset of low back pain, so we are unable to comment on causation. Second, we do not have complete medical histories on the respondents exposed to a work-related low back injury and so we are unable to identify those who had pre-existing low back problems before their injury at work. In addition, because LBP is highly prevalent in the general population [50], it can be expected that an important proportion of respondents had LBP before experiencing a work-related injury. Third, a lack of data about the severity of work-related injuries introduces uncertainty around the measures of association. For instance, even though it is likely that the group with a history of work-related low back injury mainly included respondents with mild sprain and strain injuries of the low back, it is also possible that it included respondents with severe low back injuries such as fractures and disc herniations. Since severe low back injuries may predispose a person to develop disabling low back conditions, we cannot be certain that our results do not overestimate the true associations between a lifetime history of work-related low back injury and prevalent severity-graded LBP. Finally, controlling for confounding in cross-sectional research is often difficult because we cannot be certain whether the potential confounder is a result of the exposure-outcome association or truly associated with the exposure before the outcome develops [13]. In our study, adjustment for covariates may have resulted in overadjusting and yielded conservative estimates of the associations.

## Conclusion

Our study has important implications for future research. It demonstrates the need for population-based prospective research that will investigate the causes of disabling low back pain and depression in the general population. We recommend that prognostic studies of occupational injuries use outcome measures that are able to classify the severity of the conditions of interest in a valid and reliable way. This would help avoid making conclusions potentially biased by case-mix and vague case definitions. Our analysis also highlights the importance of adequately adjusting for comorbidities and sociodemographic characteristics when studying the associations between low back injury, pain, disability, and depression. This study suggests that efforts should be devoted to investigating work-related injury as a potentially important factor in the etiology of intense and disabling LBP. The aim of this research would be to develop and implement primary and secondary prevention programs designed to decrease the burden of disabling LBP associated with work-related injuries.

In Saskatchewan, 12.3% of the adult population experiences high intensity/low disability LBP and an additional 10.7% is disabled by LBP during a 6-month period [3]. Policymakers and all relevant stakeholders should move to prevent this significant burden of disability. Finally, given the controversy about the impact of compensation and the development of pain and disability [52], there is a need to evaluate the effect of the compensation and social context on claiming behaviours and the clinical problem of work-related low back injuries.

## Competing interests

The author(s) declare that they have no competing interests.

## Authors' contributions

All authors together developed the aim and analytical approach for this report. CAH carried out the statistical analysis and wrote the first draft of the manuscript. JDC and PC contributed to the analysis and interpretation of the data and revised the manuscript for important intellectual content. All authors read and approved the final manuscript.

## Pre-publication history

The pre-publication history for this paper can be accessed here:


